# Tobacco product menthol and flavour bans: their utility for LMIC and lessons from the EU ban: Rosemary Hiscock

**DOI:** 10.1093/eurpub/ckac130.034

**Published:** 2022-10-25

**Authors:** R Hiscock, K Silver

**Affiliations:** 1 Tobacco Control Research Group, University of Bath, Bath, UK; 2 STOP Project, University of Bath, Bath, UK; 3 Tobacco Tactics, University of Bath, Bath, UK

## Abstract

**Background:**

Flavoured tobacco products increase smoking uptake and create dependence. By June 2020 all cigarettes with a characterising flavour, including menthol were banned across the EU, UK and Moldova but many low and middle income countries (LMIC) are yet to develop and implement bans despite high smoking prevalence. This paper has two objectives: to establish whether (1) flavoured tobacco products are present and marketed in LMIC and (2) the experience of bans in Europe can guide development of legislation elsewhere.

**Methods:**

Research involved analysis of menthol/flavour market data, review of academic and commercial literature and online media.

**Results:**

The median menthol/capsule market share of the cigarette market grew significantly in middle income countries (p < 0.05) between 2005 and 2019, both in lower and upper middle-income countries [lower: 2.5% (IQR: 0.5-4.0) to 6.5% (IQR: 3.6-15.9); and upper: 4.0% (IQR: 0.8-9.8) to 12.3% (IQR: 3.5-24.3)]. No market data were available on low-income countries, but the academic literature suggested high prevalence of menthol use in Zambia. Tobacco industry strategies underpinning growth of menthol/flavoured tobacco use in LMICs included in-store marketing and display, colourful packs and non-conventional flavour names. Tobacco industry attempts to circumvent the EU ban included introducing new flavoured tobacco products and accessories not included in the ban and exploiting the ban on characterising flavour (as opposed to an ingredient ban) by introducing cigarettes with lower levels of menthol.

**Conclusions:**

Banning flavoured and menthol cigarettes in LMIC would impact a growing proportion of smokers in these countries. From the European experience, menthol and flavour bans that include all tobacco products and accessories and ban flavour as an ingredient rather than a characterising flavour is recommended. Currently, lack of marketing bans and standardised packaging in LMICs exacerbate the impact of flavours.

**Key messages:**

- Flavoured and menthol tobacco products are a growing problem in LMICs.
- Bans should include all tobacco products and accessories and ban flavour as an ingredient.

